# Impact of Strategies for Preventing Obesity and Risk Factors for Eating Disorders among Adolescents: A Systematic Review

**DOI:** 10.3390/nu12103134

**Published:** 2020-10-14

**Authors:** Ana Carolina B. Leme, Jess Haines, Lisa Tang, Karin L. L. Dunker, Sonia T. Philippi, Mauro Fisberg, Gerson L. Ferrari, Regina M. Fisberg

**Affiliations:** 1 Department of Nutrition, School of Public Health, University of São Paulo, São Paulo 01246-904, Brazil; soniatphilippi@gmail.com (S.T.P.); regina.fisberg@gmail.com (R.M.F.); 2 Family Relations and Applied Nutrition, University of Guelph, Guelph, ON N1G 2W1, Canada; jhaines@uoguelph.ca (J.H.); lisa.tang@uoguelph.ca (L.T.); 3 Department of Psychiatric, Federal University of São Paulo, São Paulo 04038-000, Brazil; kdunker00@yahoo.com.br; 4 Nutrition and Feeding Difficulties Excellence Center, PENSI Institute, Sabará Children’s Hospital, São Paulo 01228-200, Brazil; mauro.fisberg@gmail.com; 5 Department of Pediatrics, Escola Paulista, Federal University of São Paulo, São Paulo 04023-062, Brazil; 6 Laboratorio de Ciencias de la Actividad Física, el Deporte y la Salud, Facultad de Ciencias Médicas, Universidad de Santiago de Chile, Santiago 8320000, Chile; gersonferrari08@yahoo.com.br

**Keywords:** obesity, eating disorders, adolescents, prevention programs, systematic review

## Abstract

An effective behavior change program is the first line of prevention for youth obesity. However, effectiveness in prevention of adolescent obesity requires several approaches, with special attention paid to disordered eating behaviors and psychological support, among other environmental factors. The aim of this systematic review is to compare the impact of two types of obesity prevention programs, inclusive of behavior change components, on weight outcomes. “Energy-balance” studies are aimed at reducing calories from high-energy sources and increasing physical activity (PA) levels, while “shared risk factors for obesity and eating disorders” focus on reducing disordered eating behaviors to promote a positive food and eating relationship. A systematic search of ProQuest, PubMed, PsycInfo, SciELO, and Web of Science identified 8825 articles. Thirty-five studies were included in the review, of which 20 regarded “energy-balance” and 15 “shared risk factors for obesity and eating disorders”. “Energy-balance” studies were unable to support maintenance weight status, diet, and PA. “Shared risk factors for obesity and eating disorders” programs also did not result in significant differences in weight status over time. However, the majority of “shared risk factors for obesity and eating disorders” studies demonstrated reduced body dissatisfaction, dieting, and weight-control behaviors. Research is needed to examine how a shared risk factor approach can address both obesity and eating disorders.

## 1. Introduction

Pediatric obesity is a well-accepted major public health concern [1]. The World Health Organization (WHO) defines pediatric obesity as a body mass index (BMI) at or above the 95th percentile among children and adolescents of the same age and sex, often measured on BMI growth charts [2]. The global age-standardized prevalence of obesity increased more than 5% for girls and almost 8% for boys over the last 40 years [2]. Causes and effects of obesity are complex and multifaceted, and obesity is associated with increased risk of these chronic conditions, such as cardiovascular diseases, type II diabetes, and certain types of cancer. However, children with obesity experience weight stigmatization, defined as the societal devaluing of an individual because of their body size [3], which often manifests in childhood as weight-based teasing and bullying [3].

Due to this stigmatization, obesity in youth has been shown to be a risk factor for psychopathology, which may manifest itself through body dissatisfaction, shape and weight concerns, and dieting and eating disorder behaviors, such as binge eating and purging [4,5]. Research has also shown that obesity in youth is associated with sneaking and hoarding food, eating when not hungry, and feelings of self-consciousness or embarrassment when eating in front of others [6,7]. Although disordered eating behaviors and eating disorders both encompass a broad array of dimensional maladaptive cognitions and behaviors relating to eating and weight, they differ in their diagnosis. The term “eating disorder” refers to a psychiatric disorder and include the following four categories: anorexia nervosa (AN), bulimia nervosa (BN), binge eating disorder (BED), and avoidant restrictive food intake disorder (ARFID) [8]. Those individuals who do not meet the specific diagnostic criteria of an eating disorder may fall into the category of a weight-related disorder, which includes disordered eating behaviors [9]. Thus, research exists to support the assessment of obesity-related problems should include disordered eating as disordered eating behaviors and obesity have similar risk factors, such as body dissatisfaction and weight control behaviors [4,10]. Eating disorders are more prevalent among those with obesity [4]. Indeed, overweight adolescents have up to five times higher odds of developing eating disorders than normal weight youth [11,12].

Prevention programs that include diet, physical activity (PA), and/or sedentary behavior components are currently the first line of prevention for obesity in adolescent youth [6]. However, focusing on diet and PA may increase the risk for eating disorders. In this approach, individuals should decrease their caloric intake and increase their levels of PA, which may encourage them to diet. Evidence has shown that the majority of individuals with eating disorders reported that they started to diet before they initiated their disordered eating behaviors [4]. The WHO Commission on Ending Childhood Obesity report [13] suggest a multi-component approach that includes comprehensive lifestyle weight-management support for youth who have an unhealthy weight status as part of a universal youth healthcare plan. Multidisciplinary prevention programs do not have a specific definition. However, the WHO report [13] noted that a comprehensive prevention plan should include psychosocial and family support in addition to common components such as nutrition and PA or sedentary behavior change. Indeed, obesity prevention programs that predominantly focus on energy-balance approaches, including diet (e.g., avoiding or choosing certain food sources) and PA (e.g., to “burn” calories), have proven to not be effective over a long period of time and may lead to an increase in the risk for disordered eating behaviors [14].

“Energy-balance” programs have a starting point on outcomes of weight gain resulting in increased caloric intake and/or decreased energy expenditure. The major components targeted are sources and amounts of foods and beverages, while energy expenditure is mainly guided by PA and metabolic rates [15]. On the other hand, “shared risk for obesity and eating disorders” programs focused on maintaining a positive relationship between food and weight through a more mindful approach in order to promote sustainable lifestyle changes [16,17].

Thus, it is important to examine the implications of the aforementioned strategies and their impact on disordered eating risk factors and obesity prevention among adolescent youth in order to build a more sustainable approach through the integration of diet and PA components with psychosocial support. Previous systematic reviews and meta-analyses [6,18] have assessed the impact of obesity treatment on eating disorders in overweight or obese children and adolescents. However, there is a gap in the literature examining the impact of obesity prevention programs among youth on risk factors for disordered eating. Thus, the aims of this systematic review are to (1) compare the impact of “energy-balance” and “shared risk factor for obesity and eating disorders” prevention programs on weight outcome changes; and (2) if the eating disorder risk factors were improved in the “shared risk factor for obesity and eating disorders” programs.

## 2. Methods

The protocol for this systematic review was registered with PROSPERO (CRD 42017076547) [10], accessible at https://www.crd.york.ac.uk/prospero/, and has been reported according to Preferred Reporting Items for Systematic Reviews and Meta-Analyses (PRISMA) guidelines [19].

### 2.1. Data Sources and Search Strategy

A systematic search of the published literature up to February 2020 was undertaken using five electronic databases, namely, PsycINFO, ProQuest, PubMed, SciElo (Scientific Electronic Library Online), and Web of Science. The following structured search strings were used: Adolescents OR Children OR Girls OR Boys OR Prevention OR Intervention AND Obesity OR Overweight OR Weight-Related Disorders OR Disordered Eating. Relevant truncations and adjacencies were used to enhance results by allowing variations of the search terms. The search was limited to studies in adolescents. Hand searching of reference lists was conducted to identify studies that may have been missed. Records were downloaded to EndNote X9.2 and duplicates removed. Records were first assessed by title and abstract and then full text. All records were assessed for inclusion based on the defined criteria. Any uncertainties regarding the inclusion of a study were resolved through discussion among A.L. and K.D. or. R.F.

### 2.2. Eligibility Criteria

All studies were assessed according to the following inclusion and exclusion criteria summarized according to PICO framework (Participants, Intervention Comparison, and Outcome):

*Participants*: Studies were eligible if they included adolescents, aged 10–19 years, as defined by the WHO [20], and inclusive of all weight statuses. Adolescents must have participated in either of the two obesity prevention programs focused on (i) energy-balance approaches (targeting diet and PA); or (ii) shared risk factors for obesity and eating disorders. All participants were eligible if they participated at the beginning of each intervention type. Participants with a pre-existing disease, an organic cause for obesity and eating disorders, or on medication that could affect weight were excluded.

*Intervention*: Energy-balance interventions were defined as an approach to improve diet (e.g., energy, fat, sugar, sodium, and fruit and vegetables intake), increase (moderate-to-vigorous) PA, and reduce screen-time with the intent to increase energy expenditure [21]. Shared risk factors for obesity and eating disorders programs were described as an approach to promote a positive relationship with weight and diet, through an improvement in the following factors that may have relevance for body image concerns: dieting, media use, body image, weight-control behaviors (e.g., use of food substitutes, diet pills, and diuretics), and maladaptive responses to weight-based teasing. These factors were selected on the basis that they are both amenable to change and suitable for addressing within prevention programs for youth [4]. Assessment of weight-related programs were obtained through the adolescents’ weight status, as well as dietary, physical activity, and sedentary behavior questionnaires. The eating disorder (ED) risk factors were obtained through a variety of different psychometric questionnaires.

*Comparison*: Different study designs (i.e., randomized controlled trials, non-randomized controlled trials, quasi-experimental control trials, and pre-post uncontrolled studies with no comparison group) were included in this review.

*Outcome*: The key outcome of interest was the impact of obesity prevention programs on disordered eating behaviors in adolescents. The outcomes assessed were related to body or shape satisfaction, weight-control behaviors, weight-teasing and/or diet intake (measured via reports), and PA levels. A secondary outcome was the impact of the programs on adolescent weight outcomes expressed as Body Mass Index (BMI), the BMI z-score (BMIz).

Excluded studies focused on the treatment of individuals with overweight or obesity. Interventions relating to the treatment of EDs or psychological morbidity were also excluded, as were studies treating secondary or syndromic causes of obesity. Search data were not time limited. No exclusion criteria were placed on intervention duration, length of follow-up, or date, but this review was limited to studies published in the English, Portuguese, and Spanish languages.

### 2.3. Data Extraction

Data were independently extracted from eligible studies by one reviewer and cross-checked for accuracy by a second reviewer. The extracted data included sample characteristics, intervention setting, intensity and design, type of studies, tools used to assess outcome measurement, and pre-, post-intervention and/or follow-up data for both types of approaches.

### 2.4. Data Synthesis

Due to the heterogeneity of the study population’s characteristics and programs features (i.e., length of treatment, outcomes measured, and timing of assessment), it was not possible to perform a meta-analysis. A narrative summary of the findings was conducted.

### 2.5. Quality Assessment and Risk of Bias

**Figure 1:**
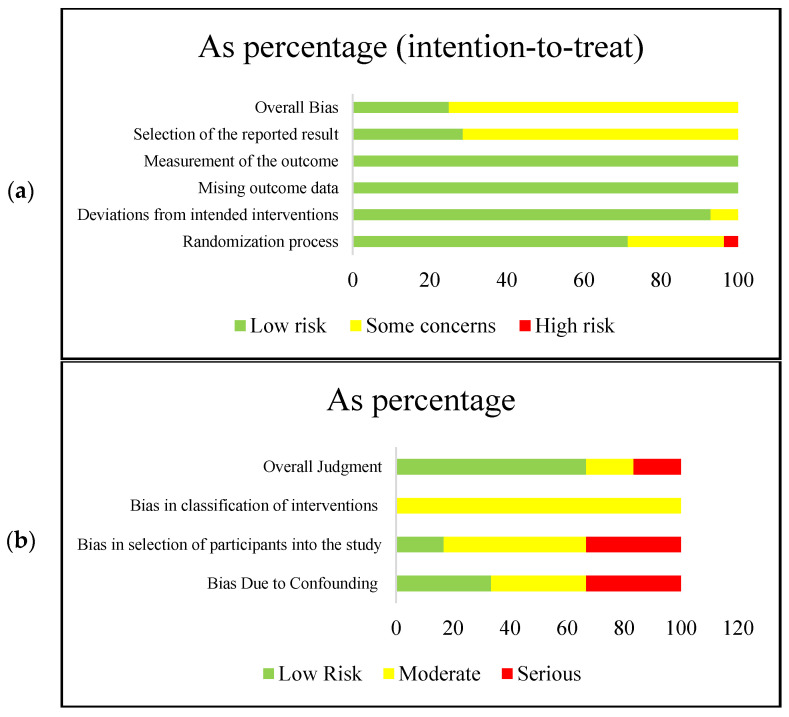
Risk of bias for randomized controlled trials (*n* = 27) (**a**) and non-randomized controlled trials (**b**). Based on the revised Cochrane Risk-of-Bias tools for randomized controlled trials (ROB-2) and non-randomized controlled trials (ROBINS-1) (*n* = 6).

Study quality was assessed using a designed appraisal tool developed by Cochrane: Version 2 of the Cochrane Risk-of-Bias tool for randomized trials (RoB 2) [22] and Risk-Of-Bias In Non-Randomized Studies (ROBINS-I tool) [23]. Individual component quality rankings, including the risk of bias measures, are included in Figure 1. Component and overall quality ratings were scored as “low risk”, “moderate”, or “high” for the RoB 2; and as “low risk”, “moderate”, and “serious” for the ROBINS-I [22,23].

## 3. Results

### 3.1. Overview of Studies

**Figure 2:**
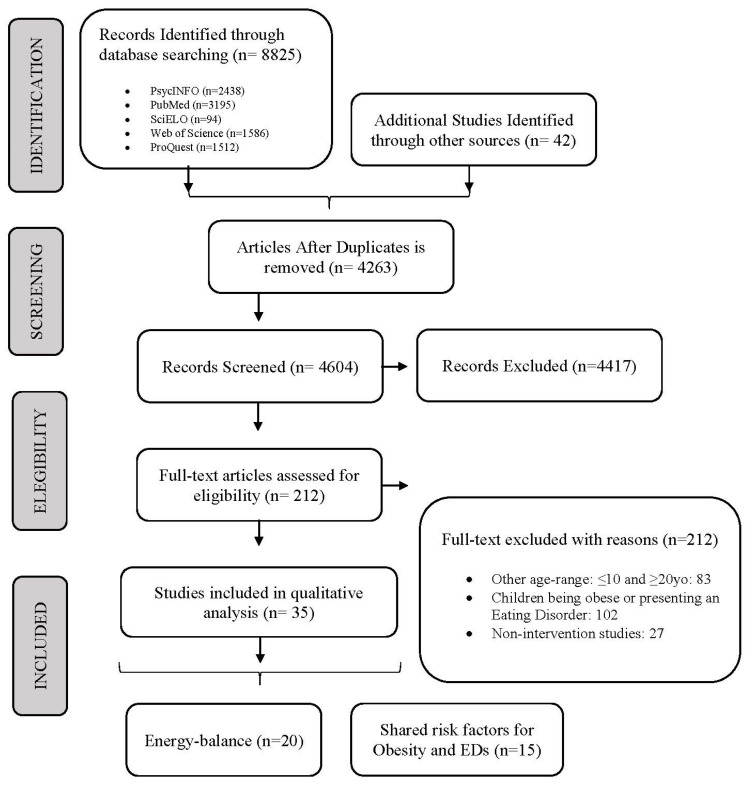
Flowchart showing the process of article selection.

A flowchart summarizing the study selection procedure is presented in Figure 2. Electronic searches returned 8825 records. To begin, duplicates (*n* = 4106) were removed. Secondly, a total of 4629 studies were screened by titles and abstracts. Finally, 211 studies were further excluded after reading through the full text. Of the excluded studies, 83 targeted children (≤10 years old) or adults (≥20 years old), 101 included non-healthy individuals (e.g., obese, eating disorders, or other health conditions), and in 28 studies an intervention was not considered. The remaining 35 studies met the inclusion criteria and were therefore eligible and included in this review. A total of 20 studies were energy-balance intervention studies, and 15 studies regarded a shared risk obesity and eating disorders program.

Data abstraction revealed 15 programs that had multiple publications; these included protocols, additional cohorts, further follow-up timepoints, different outcome measures, or other secondary analysis. Thus, for reporting and analysis, studies were grouped by program cohort. Any uncertainties regarding appropriate program cohort for categorization were resolved through discussion.

### 3.2. Study Characteristics

The characteristics of the selected studies are reported in Table 1 and divided by intervention types: “energy-balance” and “shared risk factors for obesity and eating disorders” programs. All studies were published between 2005 and 2019.

Overall, twelve studies (33.3%) were conducted in the U.S., five studies (14.7%) were conducted in Australia and other countries in Oceania [24,25,26,27,28], and three studies (8.8%) in Brazil [5,29,30] and in Spain [31,32,33]. Other studies included countries within Europe [34,35,36,37,38] and Asia [39,40,41], Canada [42], Mexico [43], and Israel [44]. Programs were evaluated as controlled trials (*n* = 31, 91.2%), randomized controlled trials (*n* = 23, 67.6%), quasi-experimental (*n* = 7, 20.5%), non-randomized (*n* = 2, 5.8%), and as interrupted time series without a comparison group (*n* = 3, 8.8%).

#### 3.2.1. Energy-Balance Programs

For the energy-balance programs, 20 studies were found [24,25,30,32,34,37,38,39,40,41,44,45,46,47,48,49,50]; of these, five studies were conducted in the USA [45,46,47,49,50], six in Europe [32,34,35,37,38], four in Australia [24,25,26,27], three in Asia [39,40,44], and one in Brazil [30] and in Tonga [41]. When considering the study design, 13 studies were randomized controlled trials (RCT) [25,26,27,30,36,37,38,40,45,46,47,49,50,51], four were quasi-experimental trials [24,34,39,41], and one non-randomized controlled trial [44]. Two studies were one-group pre- and post-test assessments [32,50]. The sample size for the energy-balance programs ranged from 51 [46] to 3638 [35] and the mean age was 12.7 ± 1.8 years old. Two targeted only females [26,46] and one only males [25]. Eight studies reported following a theoretical basis, with six studies following the Social Cognitive Theory [25,26,27,45,47,49], and two the Self-Determination Theory [25,45]. Although not reporting a theoretical framework, 10 [24,25,26,27,35,36,37,41,44,47] combined educational techniques with changes in the environment.

#### 3.2.2. Shared Risk Factors for Obesity and Eating Disorders Programs

With regards to the “shared risk factors for obesity and eating disorders” programs, fifteen studies were included in this systematic review [5,28,29,31,33,42,43,51,52,53,54,55,56,57,58]. Seven studies were conducted in the USA [51,52,54,56,57,58], two in Spain [31,33] and in Brazil [5,29], and one in Australia [28], Canada [42], and Mexico [43]. The programs included nine RCT [5,29,51,53,54,55,56,57,58], three quasi-experimental trials [31,33,43], one non-RCT [42], and one one-group pre- and post-test assessment [52]. The sample size ranged from 27 [52] to 1451 [56] adolescents participating in the shared risk factors for obesity and eating disorders program, with 15.1 ± 2.6 years old as the mean age of the participants. Six targeted only females [5,29,31,43,51,52,57]. From a theoretical approach, five was based on Social Cognitive Theory [5,29,33,51,55] and eight combined educational techniques with changes in the environment [5,29,31,42,51,56,57,58]. This was followed by focusing on approaches to reduce the risk for eating disorders, such as Media Literacy [28,31,33], Dissonance Behavioral Intervention [31,52], and Interpersonal Therapy [58].

### 3.3. The Studies’ Techniques

#### 3.3.1. Energy-Balance Programs

The majority (*n* = 13, 65%) of the energy-balance programs used schools as the main settings for integrating diet and PA for promoting behavioral changes [24,25,26,27,32,34,37,38,39,41,44,45,50], 3 (15%) use the home setting [30,47,50], and two (10%) developed a web-based platform to deliver the intervention [40,46]. Some programs, which used schools as the main setting, combined multiple components to deliver the intervention, such as combining weekly text messages and other mobile device technology (e.g., developing an app) [25,26], or integrating the family context (e.g., parents newsletters, homework, and booklets) [26,34,50].

#### 3.3.2. Shared Risk Factors for Obesity and Eating Disorders Programs

From the fourteen studies that have shared risk factors for obesity and eating disorders as the program approach, thirteen (92.3%) were conducted at school or university [5,28,29,31,33,42,43,51,52,53,54,56,57], and one was delivered through an internet-based platform [55]. Some of the school programs focused only on psychotherapy sessions to promote a healthy relationship with body and food [28,33,42,43,52,54,58], while others (*n* = 6, 42.8%) [5,29,31,51,56,57] focused on both the psychotherapy sessions and other components to help achieve a sustainable diet and PA behaviors through their life course. Other components included weekly text messages, cooking classes, enhanced physical education classes, and healthy lunches and morning snacks at school time. Two studies [29,51] used individual counseling techniques to promote intrinsic motivational for behavior change via the motivational interview technique.

### 3.4. The Studies’ Assessment Tools

#### 3.4.1. Energy-Balance Programs

Weight status was assessed in all the energy balance studies. Some of these studies (*n* = 13, 65.0%) [25,26,27,30,36,37,38,39,41,44,49,50] combined other measurements to assess anthropometric outcomes, such as body fat percentage, waist circumference, and/or waist-to-hip ratio. Dietary intake was assessed with either food frequency questionnaires [25,26,27,30,44] or 24 h recalls [34,45,46,49]. Those studies that utilized 24 h recalls used at least two records to estimate participants’ usual intake. For those studies that measured PA, some used questionnaires [24,30,34,39,45], while others used objective measurements such as accelerometers [25,26,27,49] or pedometers [34]. Some studies (*n* = 6, 30.0%) evaluated the youth’s physical fitness (e.g., cardiovascular, flexibility, muscular, strength, and agility) [25,27,36,39,45,50]. Five studies (25.0%) evaluated sedentary behaviors, with a particular focus on screen-time questionnaires [24,25,26,37,46]. Four studies (20.0%) [24,34,39,44] assessed nutrition and PA knowledge gained through the program, and four studies assessed other mental health outcomes (e.g., health-related quality of life, depressive symptomatology, or disordered eating components) [24,34,35,47]. Studies that assessed mental health outcomes were not identified as a shared risk factors for obesity and eating disorders program as they did not target these components on the intervention but rather used this as an indicator of inclusionary/exclusionary criteria of participants, and as a secondary outcome of the intervention. Three studies (15.0%) [37,39,50] used biomarkers, such as plasma glucose and lipids, as an outcome of diet and PA behavior change. Finally, two studies (10.0%) [38,47] targeted the pre-adolescent age group (10–12 years old).

#### 3.4.2. Shared Risk Factors for Obesity and Eating Disorders Programs

For the shared risk factors for obesity and eating disorders program, all of these studies evaluated participant weight status through the use of BMI. Five studies (35.7%) [5,51,54,57,58] combined this measure with other anthropometric measures, including waist circumference and % body fat (measured using DEXA, bioimpedance, or skinfolds). Seven studies (50.0%) evaluated diet intake through validated and reproduced food frequency questionnaires [5,52,54] and others through 24 h recalls [28,51,56,57]. Those studies that used a 24 h recall only collected data from one-day of diet intake. PA was assessed by seven studies: five studies [5,51,52,56,57] used at least a 3-day recall to assess the PA of the participants, while the remaining studies [28,43,54] used a validated and reproduced questionnaire (e.g., International Physical Activity Questionnaire (IPAQ) [59] and the Paffenbarg Activity Questionnaire [60]).

Measures used to evaluate the shared risk factors for obesity and eating disorders are shown in Table 1 along with the study characteristics. Ten studies (71.4%) [28,31,33,42,52,54,55,58,61] used validated and reliable measurements that are used to assess eating disorders, including the “Eating Disorder Diagnostic Screening (EDDS)” [62], “Eating Disorder Questionnaire with Instruction (EDQ-I)” [63,64], “Difficulties in Emotion Regulation Scale (DERS)” [65], “Sociocultural Attitudes Towards Appearance Scale (SATAQ)” [66], and “Perceptions Of Teasing Scale (POTS)” [67], or used measures to assess emotion regulation and positive and negative effects through the “Dutch Restrained Eating Scale (DERS)” [68] and “Positive and Negative Scale—Revised (PANAS-X)” [69]. The purpose of these scales is to evaluate the occurrence of eating disorder symptoms, disordered eating behaviors, body shape and weight satisfaction, and weight-teasing by family and friends/peers. The other five studies (35.7%) [5,42,51,56,57] assessed these measures through questionnaires used in previous surveillance studies, specifically the “Youth Risk Behavioral Surveillance System Survey (YRBSSS)” and “Project EAT”. These questionnaires assessed the risk for disordered eating behaviors, including dieting, weight control behaviors, binge eating, weight-teasing, and body/weight/shape concern.

### 3.5. Outcomes

The focus of this review was to assess the impact of obesity prevention programs on improving disordered eating behaviors and maintaining a healthy weight status among adolescents by comparing the types of interventions: energy-balance and shared risk factors for obesity and eating disorders programs (Table 2).

#### 3.5.1. Energy-Balance Programs

Ten studies [24,26,27,32,35,37,38,39,44,50,70] showed small improvements on youth weight status as measured by BMI, the BMI z-scores, or percent prevalence for being overweight/obese. A reduction was observed by a difference between groups (intervention vs. control) of at least 0.1 kg/m^2^, or by a 1.7% decrease on the prevalence of being overweight/obese from baseline to post-intervention/follow-up assessments. Five studies [25,26,34,40] did not find any significant effects on weight status change, while three studies [30,36,41] showed an increase in weight status change. For example, one study showed a large increase in overweight and obesity prevalence (10.1% and 12.6%) [41], and another study [30] showed a small increase in BMI of 0.2 kg/m^2^ for the intervention group and a decrease in % of body fat. Interestingly, a study conducted by Fulkerson et al. [47] found that although they found no significant treatment group differences in BMI z-scores post-intervention, a post-hoc stratification by pubertal onset indicated pre-pubescent youth had significantly lower BMI z-scores than their control group counterparts (ß = 0.08, 95%CI 0.01, 0.34).

Four studies (20.0%) [26,30,41,45] did not find significant differences in diet outcomes after the intervention, which included the reduction of total energy intake and improvement on the intake of certain food groups (e.g., fruit and vegetables). However, five studies (25.0%) [25,27,34,46,49] showed an improvement in the intake of sugar-sweetened beverages, as well as fruit and vegetables. Sgambato et al. [30] did not find any significant differences when evaluating diet intake using a food frequency questionnaire; however, analyses of 30% of the sample that used a 24 h recall showed a significant decrease in the intake of fruit juice (△ = −0.42 ± 0.18 serving/day) compared to the control group.

PA level was improved in four studies (20.0%) [30,34,37,49] with an average of +12.5 min/week. Two studies conducted by Lubans et al., one targeting only boys [25] and the other both sexes [27], found an improvement in physical fitness (i.e., muscular fitness and resistance training) despite finding no significant effect on PA level. The remaining four studies [26,32,45,46] found no significant effect on PA level. Shawn-Peri et al. [50] found that large classes and short physical education periods are major challenges when implementing programs. Sedentary behaviors or screen-time were improved in three studies [25,26,37]. Dewar et al. [26] showed improvement in screen-time behaviors at immediate post-intervention (after 12 months from the baseline), but at follow-up (after 24 months) found no significant differences. An average of the significant difference was −30.7 min/day.

Three studies [34,44,49] evaluated the participants’ knowledge of nutrition and PA, and found significant improvements in their knowledge. Three studies [39,49,50] assessed the biological markers to verify the improvements in lifestyle behaviors, including blood pressure and fasting capillary plasma. Significant results were found among male participants with a higher BMI and older adolescents.

#### 3.5.2. Shared Risk Factors for Obesity and Eating Disorders Programs

All 14 studies showed no significant effects on weight status post-intervention. Two programs [42,52] showed an increase in BMI and weight from post-intervention to follow-up. This increase in BMI ranged from 0.2 to 0.4 kg/m^2^, reflecting on average increase of 2.9 kg. Leme et al. [5], although not finding significant results for BMI, found that results favored the intervention group (△ = −0.26 kg/m^2^), with a lower increase in waist circumference for both groups. Female participants with high levels of anxiety demonstrated stabilization in adiposity (% body fat). Moderation analyses also indicated a stronger BMI effect for youths with initially elevated symptoms of eating disorders and higher initial BMI scores [54,55], as well as for weaker eating disorder symptoms and body image dissatisfaction [54].

Six studies [28,33,51,52,56,57] also found a reduction in several risk factors that were sustained at follow-up; specifically, eating pathology, appearance satisfaction, a thin ideal, negative affect, and emotion dysregulation. Two studies [43,55] that targeted both sexes found an interaction effect for time and group in thin idealization, but disordered eating attitudes/behaviors for females only. Leme et al. [5] and Sanchez-Carracedo et al. [31] found that results for eating disorder risk factors were in the opposite hypothesis direction, including results for appearance attitudes, eating disorders symptoms, and unhealthy weight control behaviors (e.g., skipped meals, eating very little, and fasting).

Leme et al. [5], Simpson et al. [52], and Neumark-Sztainer et al. [51] were the only studies that assessed the dietary intake, PA, and sedentary behaviors of the participants. These two studies showed an improvement in healthy eating and PA. Both showed that social support, particularly for the family, was improved after intervention along with other socio-cognitive aspects. For example, behavioral strategies for healthy eating, such as preparing meals or snacks with little fat or sugar.

## 4. Discussion

This systematic review filled a gap in the literature by assessing the impact of obesity prevention programs, with behavior change components, on weight outcomes in adolescents. Diet intervention, as either a primary goal or combined with PA, has emerged as a promising component in youth obesity prevention programs. In order to assess the impact of these programs, two approaches were compared: “energy-balance” and “shared risk factors for obesity and eating disorders”. This systematic review highlights the specific differences in the program components and outcomes that goes above and beyond simply using BMI or other anthropometric measurement as the primary outcome. This review demonstrates better anthropometric outcome measures in the “energy-balance” programs that include PA components, such as enhanced physical education classes and encouragement of behavior change activities immediately post-intervention. Upon examination of interventional studies, PA seems to be moderately to highly effective in improving body composition, especially with improving resistance exercise [25,27,50] and increasing lean mass [71]. However, a posteriori effect on BMI and body weight may not be affected by PA. In line with previous work [71], several intervention studies were unable to prove success at long-term follow-up with PA outcomes. This may be explained by the approach used in these interventions, specifically educational and behavioral, which did not produce sustainable results. As shown in the risk of bias, a meta-analysis of reviews [71] has found low methodological quality of the studies included in the meta-analysis, a high level of heterogeneity, a limited number of studies available, mixed populations of overweight and non-overweight adolescents, as well as inadequate description of the interventions.

### 4.1. Combining Diet and Physical Activity Components to Promote Sustainable Lifestyle Behaviors

Numerous programs have been evaluated in the literature; however, the most effective strategy remains to be developed. Studies that have diet as a primary component have not been shown to be sustainable over time. The combination of diet and PA was shown to be a more effective tool against preventing youth obesity than diet alone. Schools were the most common setting where these interventions were implemented. Alternatively, results derived from combining PA or a diet with a secondary component like education and/or changes in the environment, such as policies focused on food canteens and the school curriculum, were associated with smaller effect sizes or even non-significant results [71]. When integrating diet and PA via mindfulness-based approaches seem to be more effective in preventing eating disorder risk factors, such as body image concerns and weight-control behaviors, and as consequence would help maintain a healthy weight status and well-being [72].

### 4.2. Maintaining Healthy Weight through Preventing Eating Disorders Risk Factors

Population-based interventions designed to maintain a healthy weight status that focused on shared risk factors for obesity and eating disorders have been a focus of attention in the general adolescent literature over the past few years [4,10,73,74]. However, few studies have been designed to reduce the burden of these shared risk factors. Indeed, results of at least some of the prevention programs suggest that weight status should not be the main outcome when considering obesity management strategies. This is because some of the disordered eating factors, mainly dieting, can lead to unsustainable dietary practices, which can lead to unhealthy weight gain or eating disorders [4]. Tanofsky-Kraff et al. [53], via exploratory post-hoc analyses, suggested evaluating the baseline social-adjustment problems (as an index for mental health problems) [75] and anxiety as moderators of the group effect on weight gain. This study revealed that adolescents with more psychosocial difficulties initially had the most benefit with intervention programs using interpersonal techniques compared to health education techniques. Moreover, both social functioning and anxiety moderate the intervention outcomes for depressed adolescents [76], as youths with a worse baseline psychosocial functioning experience the greatest improvements in depressive symptoms if they received interpersonal interventions targeting the shared risk factors as opposed to conventional approaches. Therefore, social adjustment and anxiety are important components for weight-related prevention trials and should directly focus on improving interpersonal functioning and negative mood states.

### 4.3. Other Factors That May Be Associated with an Increased Risk for Eating Disorders and Obesity, Which May Be the Target for Behavioral Interventions

Despite an overall improvement in the disordered eating risk factors, such as body satisfaction, unhealthy weight control behaviors, and teasing, two studies [5,31] identified an increased risk for these factors at follow-up, and female adolescents tend to have a less favorable impact on these risk factors than male participants. One study reported an increased risk for skipping meals, eating too little, and fasting at 6-month follow-up [5]. Similarly, a Spanish quasi-experimental trial [31] found that, post-intervention, a drive for thinness, being negatively affected, and self-esteem were in the hypothesized direction, but socio-attitudes towards appearance and eating disorder symptoms were in the opposite direction to what was hypothesized. All outcomes were non-significant. It is uncertain if these findings differ from the typical rate of development of eating disorders or obesity that are seen within obesity prevention trials targeting adolescents, and especially adolescent girls. Nevertheless, the early signs and symptoms of these shared risk factors in adolescents attempting to lose weight may be missed, particularly when the focus is on weight loss or the adolescent remains within or above a healthy weight status [18]. These findings support previous recommendations [77] that interventionists, public health policy makers, and other behavior-change researchers should monitor for the development of or exacerbation of these combined risk factors during weight-related prevention programs.

### 4.4. Caution When Targeting Other Modifiable and Non-Modifiable Risk Factors for Weight-Prevention Programs

Research suggests that attitudes towards individuals with obesity may be reduced through the provision of non-modifiable risk factors for these specific concerns [78]. Considering this, studies that focused on causes and risk factors for obesity and eating disorders identified in this review, as well as other corroborating reviews [73,79,80], showed that biological and genetic factors played a minor role in the etiology of these shared risk factors when compared with social–cultural factors. This was confirmed in studies that include both public and health professionals [73]. Thus, the published literature seems to indicate an opportunity for change in this respect. Even if successful, prevention programs of this kind presented conflicting ethical questions [81].

### 4.5. Interventions Should Be Based on Theory and Use of Psychometric Tested Measurements

The findings demonstrated that less than half of the studies with an energy-balance approach was theory-based, with the Social Cognitive Theory (SCT) [82,83] being the most used. A review suggest that interventions aimed at changing health behaviors in adolescents should target the key constructs of the theories [84]. For example, the core construct of the SCT is self-efficacy, which is the individual confidence in personal ability to acquire a certain health behavior [85]. Research criterion and findings with adolescents suggested that these constructs are important and reliable mechanisms of dietary and PA behaviors that can be changed when using extant intervention programs. However, given the validity of the theoretical determinants of behavior change in adolescents, it is hard to provide a conclusion. This might be due to the use of less optimal measures of mediators [84]. A product-o-coefficients test may be used to examine potential mechanisms of dietary [86] and physical activity behavior change [87], and this method has been used to identify mediators even if the intervention effects are not significant.

A study that tested the social cognitive mediators of behavior change [86], although none of the action theory results were significant, showed there was a few significant conceptual theory test results; this suggests that the intervention was not successful in changing key dietary and physical activity behaviors, yet the significant conceptual theory test results demonstrated that specific social cognitive constructs predicted behavior change in this trial [86,87]. Hence, findings of this review suggested the need for further good-quality, trial-based evidence, and highlights the importance of these potential mediators in changing health behaviors [88].

### 4.6. Final Thoughts on Effective Behavioral Change Interventions for Youth

As previously known [4,89], eating disorders and obesity share similar traits, but have not been the focus in the majority of the prevention trials. Further trials combining both fields are needed to clarify the components added to promote a positive food and weight relationship. For example, adding weight-teasing and body image with weight-status measurement are necessary to examine if the “shared risk factors for obesity and eating disorders” programs demonstrate key sustainable lifestyle behaviors from each weight-related component by motivating youth under a social–cognitive approach. Establishing such components would greatly inform a more precise weight-related behavioral-change prevention programs and public health policies.

### 4.7. Strengths and Limitations

The strength of this systematic review includes the development of a comprehensive search strategy applied in order to fill the literature gap on the impact of weight-related prevention trials, maintenance of a healthy weight status, and decreasing the burden of the shared risk factors for obesity and eating disorders among adolescents. However, there are some limitations of the current review that should be noted. Despite the authors’ extensive efforts, including systematic searching of databases and manual searching of literature reference lists, it is possible that studies meeting the inclusion criteria may have been missed. Furthermore, only one author performed title, abstract, and full-text screenings. However, any uncertainties regarding study inclusion were resolved through discussion among three authors. Moreover, although strict inclusion and exclusion criteria were established, the aim of some studies included in this review was not explicitly to measure the influence of weight-related concerns on weight status and other behaviors. Thus, some outcome data was not provided in detail, limiting the conclusions able to be drawn in these instances. This review was also limited by the heterogeneity of the included studies, whereby reporting measures and outcomes were often not consistent. Finally, uncertainties on risk of bias information may be considered as a limitation. Some studies did not provide relevant information on certain sources of bias, such as allocation concealment and blinding of participants, personnel and outcome assessment, an underpowered study, and an analysis not accounting for clustering.

## 5. Conclusions

In sum, this systematic review showed that energy-balance interventions produced better results on weight outcomes when integrating physical activity associated with changes in school or other community environments. Improved disordered risk factors were seen in the shared risk factors, e.g., weight-control behaviors and shape and weight concerns, especially among overweight adolescents. However, some studies found non-significant effects or even an increased risk of these shared risk factors at the post-intervention stage among adolescent girls, suggesting that a more intensive or targeted approach may be needed for this at-risk group. These findings may suggest that efficacious approaches to support a sustainable weight status, by integrating the risk factors for eating disorders with changes in the environment, could promote healthy lifestyle behaviors, especially regarding diet and physical activity. However, more research is needed to examine how a shared risk factor approach can address both obesity and eating disorders, as well as to identify whether additional support is needed for adolescent girls.

## Figures and Tables

**Table 1:** Characteristics of the included studies, assessments, and outcomes of the intervention strategies.

Studies	Intervention Name (Country)	Study Design	Sample Characteristics	Strategy and Techniques
**Shared Risk Factors for Obesity and Eating Disorders Programs**				
Simpson et al. 2019 [52]	INSPIRE (USA)	One-group pre-post-design	27 female adolescents (M = 18.6 ± SD 1.01 years old)	Dissonance-based intervention + healthy weight + dialectical behavioral therapy.
Leme et al., 2019 [5]	Healthy Habits, Healthy Girls—Brazil (Brazil)	Randomized controlled trial with post- and 6-month	253 adolescent girls (M = 16.1 ± SE 0.1 years old); 142 in intervention group	Social Cognitive Theory.
				Achieve sustainable diet and physical activity behaviors, and decrease risk factors for eating disorders.
Castillo et al. 2019 [43]	No intervention name (Mexico)	Three-arm quasi experimental study with post and 3-month follow-up	361 adolescent girls (M = 19.78 ± 2.06 years old); 133 in experimental group; 105 in control skills group and 123 non-intervention group	Cognitive Dissonance and Constructivist Approach.
				Raise awareness to beauty standards and perpetuated by the mass media.
				Increase physical activity and healthy eating.
				Improve self-esteem, build positive self-concept, and reduce extreme perfectionism, and resolve conflicts.
Lenz and Claudino et al. 2018 [29]	Adaption of the US New Moves (Brazil)	Randomized Controlled trial with post- and 6-month follow-up.	270 adolescent girls (M = 13.4 ± 0.64 years old) with 139 in intervention group.	Social Cognitive Theory.
				Address issues related to female adolescents to promote health.
Shomaker et al. 2017 [58]	No intervention name (USA)	Randomized Controlled trial with post-intervention, 6 month and 1-year follow-up	29 pre-adolescents (M = 11.7 ± 1.6 years old) with 15 in intervention group.	Family-Based Interpersonal Therapy.
				Psycho-education on interpersonal model of loss of control-eating and general skill-building applied to improve communication, increase support, and resolve conflict between parent and child.
Sánchez-Carracedo et al. 2016 [31]	The MABIC Project (Spain)	Non-randomized controlled trial with post- and 1-year follow-up.	565 adolescent girls (M= 13.8 ± 0.5 years old) with 152 in intervention group.	Social Cognitive Theory, Media Literacy Education Approach, and Cognitive Dissonance Theory.
				Increase knowledge through sessions of the practical and relevant aspects of foods.
Wilksch et al. 2015 [28]	No intervention name (Australia)	Four-arm randomized controlled trial with post, intervention, 6-month and 12-month follow-up.	1316 adolescents (M = 13.21 ± 0.68 years old) with 269 in media smart, 347 in life smart and 225 HELPP group.	Principles of media internalization (Media Smart group).
				Principles that health is more than weight (Life Smart group).
				Principles of eating disorder risk factors of internalization of social appearance ideals and comparisons.
				Evidence principles of being interactive, avoiding psychoeducation on weight-related concerns and with multiple sessions.
Stice et al. 2013 [54]	Healthy Weight 2 (USA)	Randomized controlled trial post-, 6 month, 1-year and 2-year follow-up.	398 young adults (M = 18.4, 17–20 years old) with 192 in intervention group.	Healthy weight approach to reduce eating disorders and obesity.
				Nutrition science principles for health behavior changes.
Franko et al. 2013 [55]	BodyMojo (USA)	Randomized controlled trial with 4–6 weeks and 3-month follow-up.	65 boys (M = 15.4 ± 1.4 years old) and 113 girls (M = 15.2 ± 0.3 years old), randomized in classes.	Socio-Cognitive Theory, Health Belief Model, Theory of Planned Behavior, Transtheoretical Model.
				Internet-based program for health behavior change through technology and social engagement, offering a personalized experience, goal setting, and interactive games and videos.
Gonzalez et al. 2011 [33]	No intervention name (Spain)	Three arms quasi-experimental design with post-intervention, 6 and 30-month follow-up.	443 adolescents (M = 13.5 ± 0.4 years old) with 143 media literacy and 99 media literacy and nutrition.	Social Cognitive Theory.
				Focus on media literacy to increase nutrition awareness.
				Interactive format, sessions, and new activism and media literacy components.
				Critical thinking and promotion of health and well-being to develop resilience for sociocultural messages.
Neumark-Sztainer et al. 2010 [51]	New Moves (USA)	Randomized controlled trial with post and 9-month follow-up.	356 adolescent girls (M = 15.8 ± 1.2 years old) with 182 in intervention group.	Social Cognitive Theory and Transtheoretical Model.
				Socio-environmental, personal, and behavioral factors for changes in diet, physical activity, and weight-control behaviors.
Stock et al. 2007 [42]	Healthy Bodies (Canada)	Prospective pilot study with post-intervention.	199 adolescents (4th to 7th grade) with 128 in intervention group.	Prescribed learning outcomes from the British Columbia Minister of Health.
				3 main components of healthy living: be physical activity, eat healthy, and positive body image.
				21 lessons over the study school year.
Austin et al. 2007 [56]	The 5-2-1 go! (USA)	Randomized controlled trial with post intervention.	1451 adolescents (6th and 7th grade) with 614 in intervention group.	Learning outcomes from previous trial (Planet Girls).
				Multiple modules in schools to address nutrition and physical activity in various domains: nutrition services, physical education, and policies and environment.
Austin et al. 2005 [57]	Planet Health (USA)	Randomized controlled trial with post-and 21-month follow-up.	480 adolescent girls (M = 11.5 ± 0.7 years old) with 254 in intervention group	Social Cognitive Theory.
				Interdisciplinary curriculum with materials integrated in major subject areas and physical education classes via grade- and subject appropriate skills and competencies.
**Energy-Balance Programs**				
Sgambato et al. 2019 [30]	PAAPPAS—“Parents, Students, Community Health Agents and teachers for Healthy Eating” (Brazil)	Randomized controlled trial with post-interventions	2447 adolescents (M = 11.5 ± 1.4 years old) with 1290 in intervention group.	Family Health System.
				Reduce weight gain at school and home environments.
Aperman-Itzhak et al. 2018 [44]	No intervention name (Israel)	Controlled, non-randomized and non-blinded trial with post-intervention	373 adolescents (10–12 years old) with 187 in intervention group.	Program developed by a registered dietitian and cardiologist.
				Promote healthy eating and physical activity, integrating the head of the local council stakeholders and school teachers
Yang et al. 2017 [39]	No intervention name (South Korea)	Quasi-experimental trial with 1-year follow-up	768 adolescents (M = 11.0 ± 1.5 years old) with 418 in intervention group.	Based on pre-intervention results + personalized suggestions for improving physical strength and dietary intake.
				School-based interventions with continuation in the community.
Rerksuppaphol and Rerksuppaphol 2017 [40]	No intervention name (Thailand)	Randomized controlled trial with post-intervention.	217 adolescents (M = 10.7 ± 3.1 years old) with 111 in intervention group.	Internet-based obesity program.
				Information on health nutrition, food habits, and physical activity included in text and graphics.
				Participants collect their weight and height and interpreted their weight status.
Malakellis et al. 2017 [24]	It’s Your Move—ACT IYM (Australia)	Quasi-experimental trial with 2-year follow-up.	880 adolescents (12–16 years old) with 628 in intervention group.	ANGELO framework—identify and prioritize key determinants, considering gaps in knowledge community capacity, culturally specific needs, and current health promotion.
				Changes in school and community-based environment.
Ardic and Erdogan 2017 [34]	COPE Healthy lifestyles teen program (Turkey)	Quasi-experimental trial with post and 12-month follow-up.	100 adolescents (M = 12.8 ± 0.8 years old) with 50 in intervention group.	Adaptation of US study (COPE).
				Cognitive behavioral skill building.
				Educational information for healthy lifestyle.
Lubans et al. 2016 [25]	ATLAS Boys (Australia)	Randomized controlled trial with post, 8- and 18-month follow-up.	361 adolescent boys (M = 12.7 ± 0.5 years old) with 181 in intervention group.	Self-Determination and Social Cognitive Theory.
				Increase autonomy, competence, and relatedness to improve autonomous motivation for leisure time physical activity and school sports.
Fulkerson et al. 2015 [47]	Home Plus (USA)	Randomized controlled trial with 12- and 21-month follow-up.	149 families (children M = 10.3 ± 1.4 and; parents M = 41.6 ± 7.6 years old) with 74 families in intervention group.	Social Cognitive Theory and Social Ecological Model.
				Family changes on planning, frequency, and healthiness of family meals and snacks (limiting meals related to screen-time).
Lazorick et al. 2015 [45]	MATCH (USA)	Randomized controlled trial with post-intervention follow-up.	362 adolescents (M = 13.1 ± 0.5 years old) with 189 in intervention group.	Social Cognitive Theory and Self-Determination Theory.
				Education and behavioral curriculum (school).
				Lessons delivered in sequence of a planned manner, repeated key concepts, and applied enhance skills for healthy choices.
González-Jiménez et al. 2014 [32]	No intervention name (Spain)	One group, pre post-test design	91 adolescents (15–17 years old)	Knowledge education program to reduce weight gain.
				Three workshops on healthy eating.
				Activities during physical education classes
Grydeland et al. 2014 [38]	HEIA Study (Norway)	Randomized controlled trial with 2-month follow-up	1485 adolescents (M = 11.2 ± 0.3 years old) with 465 in intervention group.	Social Ecological Framework.
				Multiple components for health promotion to increase awareness and physical activity, and reduce screen-time.
Nollen et al. 2014 [46]	No intervention name (USA)	Randomized controlled trial with post, 8-week and 12-week follow-up.	51 adolescent girls (M = 11.3 ± 1.6 years old) with 26 in intervention group.	Mobile technology with four-week 3 modules: to improve fruit and vegetable and sugar-sweetened beverages intake and screen-time.
Dewar et al. 2013 [26]	NEAT Girls (Australia)	Randomized controlled trial with 12- and 24-month follow-up.	357 adolescent girls (M = 13.2 ± 0.5 years old) with 178 in intervention group.	Social Cognitive Theory.
				Range of strategies to promote lifestyle and lifetime physical activity, improve diet intake, and reduce time on screens.
Bonsergent et al. 2013 [35]	PRALIMAP trial (France)	Randomized Controlled trial with mid- and post-intervention follow-up.	3538 adolescents (M = 15.6 ± 0.7 years old) with 1949 in education strategy and 1589 in non-education strategy. Education was divided in environmental with 1029 and non-environmental with 920 individuals. Non-education divided in environmental with 699 and non-environmental with 890 individuals.	Personal skills were used for educational strategy, detection of weight-related problems, and proposing a care model for a screening strategy and favorable and supportive environment for environmental strategy.
				* Screening = non-education
Lubans et al. 2011 [27]	Physical Activity Leaders—PAL (Australia)	Randomized controlled trial with 3- and 6-month follow-up.	100 adolescents (M = 14.3 ± 0.6 years old) with 50 in intervention group.	Social Cognitive Theory.
				Promotion of lifestyle and lifetime activities.
Jansen et al. 2011 [36]	Lekker Fit (Enjoy being fit) (The Netherlands)	Randomized controlled trial with post-intervention.	1236 adolescents (M = 10.8 ± 1.0 years old) with 583 in intervention group.	Theory of Planned Behavior.
				ANGELO framework (identify and prioritize environmental determinants).
				Intervention targeted individual behaviors, school policies, and curriculum.
Fotu et al. 2011 [41]	Ma’alahi Youth Project (Tonga)	Quasi-experimental design with 3-year follow-up	1712 adolescents (M = 14.8 ± 1.9 years old) with 897 in intervention group.	Develop on communities the capacity to build on their own promotion for a healthy lifestyle.
				Social marketing approaches, community capacity building, and grass-roots activities.
Chen et al. 2011 [49]	WEB ABC study (USA)	Randomized controlled trial with 2-, 6- and 8-month follow-up	63 adolescents (M = 12.5 ± 3.2 years old) with 27 in intervention group	Transtheoretical Model and Social Cognitive Theory.
				Web-based program to enhance diet and physical activity self-efficacy, ease comprehension, and use problem solving skills.
Simon et al. 2008 [37]	No intervention name (France)	Randomized controlled trial with post and 4-year follow-up.	954 adolescents (M = 11.6 ± 0.6 years old) with 475 in intervention group	Multilevel theory-based.
				Provide environment institutional conditions to promote health use knowledge and skills acquired.
				Changes attitudes towards health and social support from parents and educators.
Shaw-Peri et al. 2007 [50]	NEEMA (USA)	One-group with pre-post design.	269 adolescents (M = 10.5 ± 0.7 years old)	Based on the learning outcomes of a previous study reporting increased risk for diabetes type 2.
				Changes in social structures to promote physical activity, fiber intake, and reduce saturated fat, sugar, and sedentary time.

**Table 2:** Findings from the intervention studies.

Author, Publication Year	Assessment at Follow-Up	Summary of Main Results
**Shared Risk Factors for Obesity and Eating Disorders Studies**		
Simpson et al. 2019 [52]	Eating disorders symptoms/Body shape satisfaction. Emotion regulation. Positive/negative affect. Weight status. Diet intake. Physical activity.	↓eating pathology, eating satisfaction, thin-ideal internalization, restrained eating, negative affect, emotion dysregulation. ↓ fat intake. No significant increase in BMI. Acceptable and feasible.
Leme et al. 2019 [5]	Body and shape satisfaction. Weight-control behaviors. Weight stigma. Social cognitive aspects of diet and PA. Diet intake. Physical activity. Weight status.	No significant decrease in BMI. Increase in waist circumference. Week and weekends decrease time on screens. Weekends increase vegetables intake. Social support and strategies were improved. Unhealthy weight was increased (favoring intervention group).
Castillo et al. 2019 [43]	Body and weight image. Risk factors for eating disorders. Emotion regulation. Sex-specific image concerns. Physical Activity. Weight status.	Male students did not present any significant effect. Girls improved significant for thin-ideal internalization and disordered eating attitudes.
Dunker and Claudino 2018 [29]	Body image. Emotional regulations. Weight status.	No significant results for any eating disorders risk factors. Participants’ low adherence in the program.
Shomaker et al. 2017 [58]	Weight status and body fat. Risk factors for eating disorders. Emotional regulation. Positive/negative affect.	Intervention was feasible and acceptable. Benefits to social interactions and eating. Family-based interpersonal therapy improved depression and anxiety, and loss of control compared to health education (control). Family-based interpersonal therapy reduced disordered eating attitudes. No significant differences in BMI
Sanchez-Carracedo et al. 2016 [31]	Risk for eating disorders. Body image concern. Emotional regulations. Weight status and body fat. Diet intake. Physical activity.	Media Smart and HELPP were less concerned about their shape and weight compared to control girls. Media Smart and control had less eating concerns and pressure than HELPP girls. Media Smart and HELPP benefitted from media internalization compared to control boys. Media Smart had more physical activity than HELPP and control participants. Media Smart had less time spent on screens than control participants.
Wilksch et al. 2015 [28]	Weight status Risk for eating disorders. Body image concern. Emotional regulations. Weight status and body fat. Diet intake. Physical activity.	Intervention group reduced body dissatisfaction and eating disorders symptoms. No effects for BMI, depressive symptoms, dieting, energy intake, and physical activity.
Stice et al. 2013 [54]	Risk factors for eating disorders. Body image concern. Emotion regulation.	Intervention decreased body image concerns compared to control girls (but not sustained over a 3-month follow-up). Among boys there were no significant differences between intervention and control groups.
Franko et al. 2013 [55]	Weight status. Risk factors for eating disorders. Body image concern.	Prevention presented lower risk factors for eating disorders and body image concern than the control group.
Gonzalez et al. 2011 [33]	Weight status and body fat %. Physical activity. Diet intake. Body image concern. Weight control behaviors. Social cognitive aspects of health.	No significant differences in BMI. Improvement in screen-time, diet intake, weight-control behaviors, and body image. Friends, teachers and family support for diet and physical activity behaviors.
Neumark-Sztainer et al. 2010 [51]	Weight status. Cardiovascular markers. Physical fitness. Knowledge on behavior and attitudes towards health behaviors. Emotional regulation. Body image concern. Risk for eating disorders.	BMI and weight decreased. Improvement in health knowledge: body image, eating disorders risk factors, physical activity and diet. Increase in systolic blood pressure.
Stock et al. 2007 [42]	Weight control behaviors. Diet intake. Physical activity. Weight status and body fat %.	Girls reported less weight-control behaviors after intervention. No significant differences for boys.
Austin et al. 2007 [56]	Weight control behaviors. Diet intake. Physical activity. Weight status and body fat %.	Girls reported less purging and using diet pills to control weight from both intervention and control groups.
Austin et al. 2005 [57]	Eating disorders symptoms/Body shape satisfaction. Emotion regulation. Positive/negative affect. Weight status. Diet intake. Physical activity.	↓eating pathology, eating satisfaction, thin-ideal internalization, restrained eating, negative affect, emotion dysregulation. ↓ fat intake. No significant increase in BMI. Acceptable and feasible.
**Energy-Balance Programs**		
Sgambato et al. 2019 [30]	Diet intake. Physical Activity. Health knowledge, attitudes and behaviors. Weight status and body fat %.	Weight status increased in the intervention group. Small decrease in body fat %. No significant differences on daily frequency intake of foods. Physical activity increased in the intervention group. 30% of the sample was analyzed using a 24 h Recall and significantly decrease fruit juice in the intervention group.
Aperman-Itzhak et al. 2018 [44]	Weight status, waist circumference, and body fat %. Blood pressure. Physical fitness. Health behaviors: physical activity, sleep, and diet intake. Nutrition knowledge. Body image. Emotion regulations. Parents’ obesity social-determinants aspects.	Overweight and obesity decreased only in the intervention group. Religious children have increased risk for being overweight. Knowledge improved in the intervention and control groups.
Yang et al. 2017 [39]	Weight status, body fat %. Blood pressure. Physical fitness.	No significant difference in overweight incidence between the intervention and control groups. Intervention decreased BMI, height, body fat %, and increased muscular fitness compared to the control group. Blood pressure was significantly reduced, mainly in those with higher BMI, boys, and older children. Physical fitness was improved. Normal weight boys and younger individuals showed better weight-related outcomes.
Rerksuppaphol and Rerksuppaphol 2017 [40]	Weight status.	Control showed an increased in overweight and BMI compared to the intervention group.
Malakellis et al. 2017 [24]	Weight status. Health knowledge, attitudes and behaviors. Environment perceptions (home, school, and neighborhood). Emotional regulations.	Two of three intervention schools decreased the prevalence of overweight.
Ardic and Erdocan 2017 [34]	Weight status. Physical activity (daily steps). Diet and water intake. Nutrition and physical activity knowledge. Emotional regulations.	Intervention group improve diet, physical activity, and stress management. Increased number of daily steps/weeks, fruit and vegetables, and water intake. Knowledge about nutrition and physical activity was improved. Anxiety levels and BMI were reduced, but effects were not significant.
Lubans et al. 2016 [25]	Weight status and waist circumference. Physical activity and sedentary behaviors. Sugar-sweetened beverages intake. Muscular fitness and resistance training skills. School sports motivation regulation.	No significant effect for BMI, waist circumference, and body fat %. No significant effect for physical activity. Screen-time, sugar-sweetened beverages, muscular fitness, and resistance training were improved.
Lazorick et al. 2015 [45]	Weight status. Physical fitness. Diet intake. Physical activity and sedentary behaviors. Sleep behaviors.	MATCH significant decreased BMI compared to the control group. Subgroup analysis showed decreased among overweight and obese participants. Lifestyle behaviors were not significant.
Fulkerson et al. 2015 [47]	Weight status. Pubertal development scale. Family dinner frequency.	No significant difference in BMI; but promising reduction in excess weight gain. Subgroup analysis showed that pre-pubescent children showed lower BMI in the intervention group.
Gonzalez-Jimenez et al. 2014 [32]	Weight status, waist circumference, and waist-to-hip ratio. Pubertal category scores	Weight status was improved. Significant results for diet intake. No significant results for physical activity.
Nollen et al. 2014 [46]	Home availability of fruit and vegetables, sugar-sweetened beverages, and screen devices. Diet intake. Screen-time behaviors.	Mobile technology used the program about 63% of days compared to the control girls. Non-significant increase in fruit and vegetables and decrease in sugar-sweetened beverage intake. No significant differences for BMI and screen-time use.
Dewar et al. 2013 [26]	Weight status and body fat %. Physical activity and sedentary behaviors. Diet intake.	Non-significant effect on the decrease for BMI and body fat % between the intervention and control groups. Screen-time was significantly reduced. No significant effect for physical activity, diet intake, and self-esteem.
Bonsergent et al. 2013 [35]	Weight status. Emotional regulations. Risk factors for eating disorders.	Screening improved the BMI and decreased the overweight incidence compared to the non-screening strategy. Education and environment strategies were less effective.
Lubans et al. 2011 [27]	Weight status, body fat %, and waist circumference. Physical fitness. Physical activity. Fruit and vegetables, sugar-sweetened beverages, and water intake.	Significant effect in BMI and body fat %. No significant effect for waist circumference, muscular fitness, and physical activity. Adolescents reported less intake on sugar-sweetened beverages after intervention.
Jansen et al. 2011 [36]	Weight status and waist circumference. Physical fitness.	Overweight increased at both the intervention and control groups. No significant effects for BMI.
Fotu et al. 2011 [41]	Weight status and body fat %. Diet intake. Physical activity.	Increased in overweight prevalence. Intervention group decrease body fat %. Diet and physical activity were not improved.
Chen et al. 2011 [49]	Weight status and waist-to-hip ratio. Blood pressure. Diet and physical activity knowledge and self-efficacy. Diet intake. Physical activity.	Waist-to-hip ratio and diastolic blood pressure were decreased. Fruit and vegetables intake, and physical activity were improved. Nutrition and physical activity knowledge improved.
Grydeland et al. 2014 [38]	Weight status, waist circumference, and waist-to-hip circumference.	Effects on BMI only for girls. Beneficial effect for BMI in participants with high educated parents. Negative effects for waist-to-hip ratio in participants with low educated parents. No significant for waist circumference and weight status.
Simon et al. 2008 [37]	Weight status. Physical activity. Plasma lipids.	Intervention lower increased in BMI than control groups. Intervention better effect on non-overweight students. Non-significant differences in overweight students. Intervention improved supervised PA, screen-time, and HDL-c.
Shaw-Peri et al. 2007 [51]	Weight status and % body fat. Plasma glucose.	Fitness laps, fasting glucose, and % body fat improved by the end of the study.

## References

[1] Rome E.S. (2011). Obesity prevention and treatment. Pediatr. Rev..

[2] NCD Risk Factor Collaboration (NCD-RisC) (2017). Worldwide trends in body-mass index, underweight, overweight, and obesity from 1975 to 2016: A pooled analysis of 2416 population-based measurement studies in 128.9 million children, adolescents, and adults. Lancet.

[3] Pont S.J., Puhl R., Cook S.R., Slusser W. (2017). Stigma experienced by children and adolescents with obesity. Pediatrics.

[4] Haines J., Neumark-Sztainer D. (2006). Prevention of obesity and eating disorders: A consideration of shared risk factors. Health Educ. Res..

[5] Leme A.C.B., Philippi S.T., Thompson D., Nicklas T., Baranowski T. (2019). “Healthy habits, healthy girls-Brazil”: An obesity prevention program with added focus on eating disorders. Eat. Weight Disord. EWD.

[6] De Giuseppe R., Di Napoli I., Porri D., Cena H. (2019). Pediatric obesity and eating disorders symptoms: The role of the multidisciplinary treatment. A systematic review. Front. Pediatr..

[7] Fiechtner L., Fonte M.L., Castro I., Gerber M., Horan C., Sharifi M., Cena H., Taveras E.M. (2018). Determinants of binge eating symptoms in children with overweight/obesity. Child. Obes..

[8] Swanson S.A., Crow S.J., Le Grange D., Swendsen J., Merikangas K.R. (2011). Prevalence and correlates of eating disorders in adolescents. Results from the national comorbidity survey replication adolescent supplement. Arch. Gen. Psychiatry.

[9] Murray S.B., Griffiths S., Mond J.M. (2016). Evolving eating disorder psychopathology: Conceptualising muscularity-oriented disordered eating. Br. J. Psychiatry.

[10] Leme A.C.B., Thompson D., Lenz Dunker K.L., Nicklas T., Tucunduva Philippi S., Lopez T., Vezina-Im L.A., Baranowski T. (2018). Obesity and eating disorders in integrative prevention programmes for adolescents: Protocol for a systematic review and meta-analysis. BMJ Open.

[11] Evans E.H., Adamson A.J., Basterfield L., Le Couteur A., Reilly J.K., Reilly J.J., Parkinson K.N. (2017). Risk factors for eating disorder symptoms at 12 years of age: A 6-year longitudinal cohort study. Appetite.

[12] Veses A.M., Martínez-Gómez D., Gómez-Martínez S., Vicente-Rodriguez G., Castillo R., Ortega F.B., González-Gross M., Calle M.E., Veiga O.L., Marcos A. (2014). Physical fitness, overweight and the risk of eating disorders in adolescents. The AVENA and AFINOS studies. Pediatr. Obes..

[13] World Health Organization (2017). Report of the Commission on Ending Childhood Obesity: Implementation Plan: Executive Summary.

[14] Rubino F., Puhl R.M., Cummings D.E., Eckel R.H., Ryan D.H., Mechanick J.I., Nadglowski J., Ramos Salas X., Schauer P.R., Twenefour D. (2020). Joint international consensus statement for ending stigma of obesity. Nat. Med..

[15] Pegington M., French D.P., Harvie M.N. (2020). Why young women gain weight: A narrative review of influencing factors and possible solutions. Obes. Rev. Off. J. Int. Assoc. Study Obes..

[16] Neumark-Sztainer D. (2012). Integrating messages from the eating disorders field into obesity prevention. Adolesc. Med. State Art Rev..

[17] Neumark-Sztainer D., Levine M.P., Paxton S.J., Smolak L., Piran N., Wertheim E.H. (2006). Prevention of body dissatisfaction and disordered eating: What next?. Eat. Disord..

[18] Jebeile H., Gow M.L., Baur L.A., Garnett S.P., Paxton S.J., Lister N.B. (2019). Treatment of obesity, with a dietary component, and eating disorder risk in children and adolescents: A systematic review with meta-analysis. Obes Rev..

[19] Moher D., Liberati A., Tetzlaff J., Altman D.G., The P.G. (2009). Preferred Reporting items for systematic reviews and meta-analyses: The PRISMA statement. PLoS Med..

[20] World Health Organization (WHO) Health Topics—*Adolescent Health*. https://www.who.int/health-topics/adolescent-health#tab=tab_1.

[21] Schoenberg N.E., Tarasenko Y.N., Snell-Rood C. (2018). Are evidence-based, community-engaged energy balance interventions enough for extremely vulnerable populations?. Transl. Behav. Med..

[22] Sterne J.A.C., Savović J., Page M.J., Elbers R.G., Blencowe N.S., Boutron I., Cates C.J., Cheng H.-Y., Corbett M.S., Eldridge S.M. (2019). RoB 2: A revised tool for assessing risk of bias in randomised trials. BMJ (Clin. Res. Ed.).

[23] Sterne J.A.C., Hernán M.A., Reeves B.C., Savović J., Berkman N.D., Viswanathan M., Henry D., Altman D.G., Ansari M.T., Boutron I. (2016). ROBINS-I: A tool for assessing risk of bias in non-randomised studies of interventions. BMJ (Clin. Res. Ed.).

[24] Malakellis M., Hoare E., Sanigorski A., Crooks N., Allender S., Nichols M., Swinburn B., Chikwendu C., Kelly P.M., Petersen S. (2017). School-Based systems change for obesity prevention in adolescents: Outcomes of the Australian capital territory “It’s Your Move!”. Aust. N. Z. J. Public Health.

[25] Lubans D.R., Smith J.J., Plotnikoff R.C., Dally K.A., Okely A.D., Salmon J., Morgan P.J. (2016). Assessing the sustained impact of a school-based obesity prevention program for adolescent boys: The ATLAS cluster randomized controlled trial. Int. J. Behav. Nutr. Phys. Act..

[26] Dewar D.L., Morgan P.J., Plotnikoff R.C., Okely A.D., Collins C.E., Batterham M., Callister R., Lubans D.R. (2013). The nutrition and enjoyable activity for teen girls study: A cluster randomized controlled trial. Am. J. Prev. Med..

[27] Lubans D.R., Morgan P.J., Aguiar E.J., Callister R. (2011). Randomized controlled trial of the physical activity leaders (PALs) program for adolescent boys from disadvantaged secondary schools. Prev. Med. Int. J. Devoted Pract. Theory.

[28] Wilksch S.M., Paxton S.J., Byrne S.M., Austin S.B., McLean S.A., Thompson K.M., Dorairaj K., Wade T.D. (2015). Prevention across the spectrum: A randomized controlled trial of three programs to reduce risk factors for both eating disorders and obesity. Psychol. Med..

[29] Lenz Dunker K.L., Claudino A.M. (2018). Preventing weight-related problems among adolescent girls: A cluster randomized trial comparing the Brazilian ‘New Moves’ program versus observation. Obes. Res. Clin. Pract..

[30] Sgambato M.R., Cunha D.B., da Silva Nalin Souza B., Henriques V.T., da Rocha Muniz Rodrigues R., Viegas Rego A.L., Pereira R.A., Yokoo E.M., Sichieri R. (2019). Effectiveness of school-home intervention for adolescent obesity prevention: Parallel school-randomized study. Br. J. Nutr..

[31] Sánchez-Carracedo D., Fauquet J., López-Guimerà G., Leiva D., Puntí J., Trepat E., Pàmias M., Palao D. (2016). The MABIC project: An effectiveness trial for reducing risk factors for eating disorders. Behav. Res. Ther..

[32] González-Jiménez E., Cañadas G.R., Lastra-Caro A., Cañadas-De la Fuente G.A. (2014). Efectividad de una intervención educativa sobre nutrición y actividad física en una población de adolescentes: Prevención de factores de riesgos endocrino-metabólicos y cardiovasculares. Aquichan.

[33] Gonzalez M., Penelo E., Gutierrez T., Raich R.M. (2011). Disordered eating prevention programme in schools: A 30-month follow-up. Eur. Eat. Disord. Rev..

[34] Ardic A., Erdogan S. (2017). The effectiveness of the COPE healthy lifestyles TEEN program: A school-based intervention in middle school adolescents with 12-month follow-up. J. Adv. Nurs..

[35] Bonsergent E., Agrinier N., Thilly N., Tessier S., Legrand K., Lecomte E., Aptel E., Hercberg S., Collin J.-F., Briançon S. (2013). Overweight and obesity prevention for adolescents: A cluster randomized controlled trial in a school setting. Am. J. Prev. Med..

[36] Jansen W., Borsboom G., Meima A., Joosten-Van Zwanenburg E., Mackenbach J.P., Raat H., Brug J. (2011). Effectiveness of a primary school-based intervention to reduce overweight. Int. J. Pediatr. Obes..

[37] Simon C., Schweitzer B., Oujaa M., Wagner A., Arveiler D., Triby E., Copin N., Blanc S., Platat C. (2008). Successful overweight prevention in adolescents by increasing physical activity: A 4-year randomized controlled intervention. Int. J. Obes..

[38] Grydeland M., Bjelland M., Anderssen S.A., Klepp K.-I., Bergh I.H., Andersen L.F., Ommundsen Y., Lien N. (2014). Effects of a 20-month cluster randomised controlled school-based intervention trial on BMI of school-aged boys and girls: The HEIA study. Br. J. Sport. Med..

[39] Yang Y., Kang B., Lee E.Y., Yang H.K., Kim H.S., Lim S.Y., Lee J.H., Lee S.S., Suh B.K., Yoon K.H. (2017). Effect of an obesity prevention program focused on motivating environments in childhood: A school-based prospective study. Int. J. Obes..

[40] Rerksuppaphol L., Rerksuppaphol S. (2017). Internet based obesity prevention program for Thai school children—A randomized control trial. J. Clin. Diagn. Res..

[41] Fotu K.F., Millar L., Mavoa H., Kremer P., Moodie M., Snowdon W., Utter J., Vivili P., Schultz J.T., Malakellis M. (2011). Outcome results for the Ma’alahi Youth Project, a Tongan community-based obesity prevention programme for adolescents. Obes. Rev..

[42] Stock S., Miranda C., Evans S., Plessis S., Ridley J., Yeh S., Chanoine J.-P. (2007). Healthy buddies: A novel, peer-led health promotion program for the prevention of obesity and eating disorders in children in elementary school. Pediatrics.

[43] Castillo I., Solano S., Sepulveda A.R. (2019). A controlled study of an integrated prevention program for improving disordered eating and body image among Mexican university students: A 3-month follow-up. Eur. Eat. Disord. Rev..

[44] Aperman-Itzhak T., Yom-Tov A., Vered Z., Waysberg R., Livne I., Eilat-Adar S. (2018). School-Based intervention to promote a healthy lifestyle and obesity prevention among fifth- and sixth-grade children. Am. J. Health Educ..

[45] Lazorick S., Fang X., Hardison G.T., Crawford Y. (2015). Improved body mass index measures following a middle school-based obesity intervention-the MATCH program. J. Sch. Health.

[46] Nollen N.L., Mayo M.S., Carlson S.E., Rapoff M.A., Goggin K.J., Ellerbeck E.F. (2014). Mobile technology for obesity prevention: A randomized pilot study in racial- and ethnic-minority girls. Am. J. Prev. Med..

[47] Fulkerson J.A., Friend S., Flattum C., Horning M., Draxten M., Neumark-Sztainer D., Gurvich O., Story M., Garwick A., Kubik M.Y. (2015). Promoting healthful family meals to prevent obesity: HOME Plus, a randomized controlled trial. Int. J. Behav. Nutr. Phys. Act..

[48] Smith J.J.B., Morgan P.J.P., Plotnikoff R.C.P., Dally K.A.P., Salmon J.P., Okely A.D.P., Finn T.L., Lubans D.R.P. (2014). Smart-Phone obesity prevention trial for adolescent boys in low-income communities: The ATLAS RCT. Pediatrics.

[49] Chen J.-L., Weiss S., Heyman M.B., Cooper B., Lustig R.H. (2011). The efficacy of the web-based childhood obesity prevention program in Chinese American adolescents (Web ABC study). J. Adolesc. Health.

[50] Shaw-Perry M., Horner C., Treviño R.P., Sosa E.T., Hernandez I., Bhardwaj A. (2007). NEEMA: A school-based diabetes risk prevention program designed for African-American children. J. Natl. Med. Assoc..

[51] Neumark-Sztainer D.R., Friend S.E., Flattum C.F., Hannan P.J., Story M.T., Bauer K.W., Feldman S.B., Petrich C.A. (2010). New moves-preventing weight-related problems in adolescent girls a group-randomized study. Am. J. Prev. Med..

[52] Simpson C.C., Burnette C.B., Mazzeo S.E. (2019). Integrating eating disorder and weight gain prevention: A pilot and feasibility trial of INSPIRE. Eat. Weight Disord. EWD.

[53] Tanofsky-Kraff M., Shomaker L.B., Wilfley D.E., Young J.F., Sbrocco T., Stephens M., Brady S.M., Galescu O., Demidowich A., Olsen C.H. (2017). Excess weight gain prevention in adolescents: Three-year outcome following a randomized controlled trial. J. Consult. Clin. Psychol..

[54] Stice E., Rohde P., Shaw H., Marti C.N. (2013). Efficacy trial of a selective prevention program targeting both eating disorders and obesity among female college students: 1- and 2-year follow-up effects. J. Consult. Clin. Psychol..

[55] Franko D.L., Cousineau T.M., Rodgers R.F., Roehrig J.P. (2013). BodiMojo: Effective Internet-based promotion of positive body image in adolescent girls. Body Image.

[56] Austin S.B., Kim J., Wiecha J., Troped P.J., Feldman H.A., Peterson K.E. (2007). School-Based overweight preventive intervention lowers incidence of disordered weight-control behaviors in early adolescent girls. Arch. Pediatr. Adolesc. Med..

[57] Austin S.B., Field A.E., Wiecha J., Peterson K.E., Gortmaker S.L. (2005). The impact of a school-based obesity prevention trial on disordered weight-control behaviors in early adolescent girls. Arch. Pediatr. Adolesc. Med..

[58] Shomaker L.B., Tanofsky-Kraff M., Matherne C.E., Mehari R.D., Olsen C.H., Marwitz S.E., Bakalar J.L., Ranzenhofer L.M., Kelly N.R., Schvey N.A. (2017). A randomized, comparative pilot trial of family-based interpersonal psychotherapy for reducing psychosocial symptoms, disordered-eating, and excess weight gain in at-risk preadolescents with loss-of-control-eating. Int. J. Eat. Disord..

[59] Lee P.H., Macfarlane D.J., Lam T.H., Stewart S.M. (2011). Validity of the International Physical Activity Questionnaire Short Form (IPAQ-SF): A systematic review. Int. J. Behav. Nutr. Phys. Act..

[60] Simpson K., Parker B., Capizzi J., Thompson P., Clarkson P., Freedson P., Pescatello L.S. (2015). Validity and reliability question 8 of the Paffenbarger Physical Activity Questionnaire among healthy adults. J. Phys. Act. Health.

[61] Trott M., Jackson S.E., Firth J., Jacob L., Grabovac I., Mistry A., Stubbs B., Smith L. (2020). A comparative meta-analysis of the prevalence of exercise addiction in adults with and without indicated eating disorders. Eat. Weight Disord..

[62] Stice E., Fisher M., Martinez E. (2004). Eating disorder diagnostic scale: Additional evidence of reliability and validity. Psychol. Assess..

[63] Goldfein J.A., Devlin M.J., Kamenetz C. (2005). Eating Disorder Examination-Questionnaire with and without instruction to assess binge eating in patients with binge eating disorder. Int. J. Eat. Disord..

[64] Fairburn C.G., Beglin S.J. (1994). Assessment of eating disorders: Interview or self-report questionnaire?. Int. J. Eat. Disord..

[65] Gratz K.L., Roemer L. (2004). Multidimensional assessment of emotion regulation and dysregulation: Development, factor structure, and initial validation of the difficulties in emotion regulation scale. J. Psychopathol. Behav. Assess..

[66] Thompson J.K., van den Berg P., Roehrig M., Guarda A.S., Heinberg L.J. (2004). The sociocultural attitudes towards appearance scale-3 (SATAQ-3): Development and validation. Int. J. Eat. Disord..

[67] Thompson J.K., Cattarin J., Fowler B., Fisher E. (1995). The Perception of Teasing Scale (POTS): A revision and extension of the Physical Appearance Related Teasing Scale (PARTS). J. Personal. Assess..

[68] Van Strien T., Frijters J.E.R., van Staveren W.A., Defares P.B., Deurenberg P. (1986). The predictive validity of the Dutch Restrained Eating Scale. Int. J. Eat. Disord..

[69] Watson D., Clark L.A. (1996). Affects Separable and Inseparable: On the Hierarchical Arrangement of the Negative Affects. J. Personal. Soc. Psychol..

[70] Lacey J., Lomax A.J., McNeil C., Marthick M., Levy D., Kao S., Nielsen T., Dhillon H.M. (2019). A supportive care intervention for people with metastatic melanoma being treated with immunotherapy: A pilot study assessing feasibility, perceived benefit, and acceptability. Support. Care Cancer.

[71] Psaltopoulou T., Tzanninis S., Ntanasis-Stathopoulos I., Panotopoulos G., Kostopoulou M., Tzanninis I.G., Tsagianni A., Sergentanis T.N. (2019). Prevention and treatment of childhood and adolescent obesity: A systematic review of meta-analyses. World J. Pediatr..

[72] Morillo Sarto H., Barcelo-Soler A., Herrera-Mercadal P., Pantilie B., Navarro-Gil M., Garcia-Campayo J., Montero-Marin J. (2019). Efficacy of a mindful-eating programme to reduce emotional eating in patients suffering from overweight or obesity in primary care settings: A cluster-randomised trial protocol. BMJ Open.

[73] Bullivant B., Rhydderch S., Griffiths S., Mitchison D., Mond J.M. (2020). Eating disorders “mental health literacy”: A scoping review. J. Ment. Health.

[74] Philippi S.T., Leme A.C.B. (2018). Weight-Teasing: Does body dissatisfaction mediate weight-control behaviors of Brazilian adolescent girls from low-income communities?. Cad. Saude Publica.

[75] Rzepa S., Weissman M. (2014). Social Adjustment Scale Self-Report (SAS-SR). Encycl. Qual. Life Well-Being Res..

[76] Gunlicks-Stoessel M., Mufson L., Jekal A., Turner J.B. (2010). The impact of perceived interpersonal functioning on treatment for adolescent depression: IPT-A versus treatment as usual in school-based health clinics. J. Consult. Clin. Psychol..

[77] Goldschmidt A.B., Aspen V.P., Sinton M.M., Tanofsky-Kraff M., Wilfley D.E. (2008). Disordered eating attitudes and behaviors in overweight youth. Obesity.

[78] Pena-Romero A.C., Navas-Carrillo D., Marin F., Orenes-Pinero E. (2018). The future of nutrition: Nutrigenomics and nutrigenetics in obesity and cardiovascular diseases. Crit. Rev. Food Sci. Nutr..

[79] Hauck C., Cook B., Ellrott T. (2020). Food addiction, eating addiction and eating disorders. Proc. Nutr. Soc..

[80] Wu X.Y., Yin W.Q., Sun H.W., Yang S.X., Li X.Y., Liu H.Q. (2019). The association between disordered eating and health-related quality of life among children and adolescents: A systematic review of population-based studies. PLoS ONE.

[81] Andersson M.A., Harkness S.K. (2017). When do biological attributions of mental illness reduce stigma? Using qualitative comparative analysis to contextualize attributions. Soc. Ment. Health.

[82] Bandura A. (1986). Social Foundations of Thought and Action: A Social Cognitive Theory.

[83] Sittig S., McGowan A., Iyengar S. (2020). Extensive review of persuasive system design categories and principles: Behavioral obesity interventions. J. Med. Syst..

[84] Neumark-Sztainer D., Story M., Hannan P.J., Rex J. (2003). New Moves: A school-based obesity prevention program for adolescent girls. Prev. Med..

[85] Leme A.C., Philippi S.T. (2014). Cultural adaptation and psychometric properties of social cognitive scales related to adolescent dietary behaviors. Cad. Saúde Coletiva.

[86] McCabe B.E., Plotnikoff R.C., Dewar D.L., Collins C.E., Lubans D.R. (2015). Social cognitive mediators of dietary behavior change in adolescent girls. Am. J. Health Behav..

[87] Dewar D.L., Plotnikoff R.C., Morgan P.J., Okely A.D., Costigan S.A., Lubans D.R. (2013). Testing social-cognitive theory to explain physical activity change in adolescent girls from low-income communities. Res. Q. Exerc. Sport.

[88] Cerin E., Barnett A., Baranowski T. (2009). Testing theories of dietary behavior change in youth using the mediating variable model with intervention programs. J. Nutr. Educ. Behav..

[89] Irving L.M., Neumark-Sztainer D. (2002). Integrating the prevention of eating disorders and obesity: Feasible or futile?. Prev. Med..

